# Distinct Bacterial Communities Associated with Massive and Branching Scleractinian Corals and Potential Linkages to Coral Susceptibility to Thermal or Cold Stress

**DOI:** 10.3389/fmicb.2017.00979

**Published:** 2017-06-08

**Authors:** Jiayuan Liang, Kefu Yu, Yinghui Wang, Xueyong Huang, Wen Huang, Zhenjun Qin, Ziliang Pan, Qiucui Yao, Wenhuan Wang, Zhengchao Wu

**Affiliations:** ^1^Coral Reef Research Center of China, Guangxi UniversityNanning, China; ^2^Guangxi Laboratory on the Study of Coral Reefs in the South China SeaNanning, China; ^3^School of Marine Sciences, Guangxi UniversityNanning, China; ^4^State Key Laboratory of Tropical Oceanography, South China Sea Institute of Oceanology, Chinese Academy of SciencesGuangzhou, China

**Keywords:** coral-associated bacteria, coral bleaching, thermal or cold stress susceptibility, skeletal morphology, South China Sea

## Abstract

It is well known that different coral species have different tolerances to thermal or cold stress, which is presumed to be related to the density of *Symbiodinium*. However, the intrinsic factors between stress-tolerant characteristics and coral-associated bacteria are rarely studied. In this study, 16 massive coral and 9 branching coral colonies from 6 families, 10 genera, and 18 species were collected at the same time and location (Xinyi Reef) in the South China Sea to investigate the bacterial communities. The results of an alpha diversity analysis showed that bacterial diversities associated with massive corals were generally higher than those with branching corals at different taxonomic levels (phylum, class, order, and so on). In addition, hierarchical clustering tree and PCoA analyses showed that coral species were clustered into two large groups according to the similarity of bacterial communities. Group I consisted of massive *Goniastrea*, *Plesiastrea*, *Leptastrea*, *Platygyra*, *Echinopora*, *Porites*, and *Leptoria*, and group II consisted of branching *Acropora* and *Pocillopora*. These findings suggested that both massive corals and branching corals have their own preference for the choice of associated bacteria, which may be involved in observed differences in thermal/cold tolerances. Further analysis found that 55 bacterial phyla, including 43 formally described phyla and 12 candidate phyla, were detected in these coral species. Among them, 52 phyla were recovered from the massive coral group, and 46 phyla were recovered from the branching coral group. Formally described coral pathogens have not been detected in these coral species, suggesting that they are less likely to be threatened by disease in this geographic area. This study highlights a clear relationship between the high complexity of bacterial community associated with coral, skeletal morphology of coral and potentially tolerances to thermal or cold stress.

## Introduction

Coral-associated microorganisms, including bacteria, fungi, Archaea, dinoflagellates, eukaryotic viruses and phage (Wegley et al., 2007), play significant roles in the biogeochemical cycle, material transformation and maintaining health of coral reef ecosystems (Lesser et al., 2004; Fiore et al., 2010; Mao-Jones et al., 2010; Mahmoud and Kalendar, 2016). Among them, bacteria are the most complex group in terms of species, function, and variability (Wegley et al., 2007; Li et al., 2013; Glasl et al., 2016). Recently, studies have shown that the diversities of bacterial communities associated with corals are extremely high (Li et al., 2013), and significantly affected by the factors including species (Hong et al., 2009), geography (McKew et al., 2012), season (Chen et al., 2011; Li et al., 2014), and more (Bourne et al., 2008; Ceh et al., 2012). For example, the communities of coral-associated bacteria from the Caribbean were significantly more diverse than their Indonesian counterparts and some of the coral host-specific communities (e.g., *Clostridiales* associated with *Acropora* spp.) were distinct (McKew et al., 2012). Bacterial communities associated with *Acropora tenuis*, *Tubastrea faulkneri*, and *Pocillopora damicornis* were also analyzed before and after mass spawning on Ningaloo Reef in Western Australia. The results indicated that bacterial diversity increased for all coral species after spawning; some bacterial groups (e.g., *Roseobacter*, *Erythrobacter*, and *Alteromonadales*) that may play an important role in coral reproduction were found to be prominent in these coral species (Ceh et al., 2012). Bacterial communities associated with *Porites lutea* from Luhuitou fringing reef located in Sanya, southern Hainan Island, northern South China Sea, had a much more dynamic seasonal response and were also significantly different between mucus, tissue and skeleton (Li et al., 2014). In addition, Meron et al. (2011) further showed that ocean acidification would affect the community structure of coral-associated bacteria, and some potential pathogens, such as *Vibrionaceae* and *Alteromonadaceae*, would become active with an increasing abundance.

With the development of DNA sequencing technology, an increasing number of bacterial phyla (including some candidate phyla) associated with corals have been identified. Overall, the dominant bacterial phyla were similar among different coral species, of which *Proteobacteria*, *Bacteroidetes*, *Firmicutes*, *Cyanobacteria*, and *Chloroflexi* were the most predominant bacterial groups with an abundance of over 90% (Meron et al., 2011; Li et al., 2014). In addition, many candidate phyla (*WS3*, *BRC1*, *OD1*, and so on) were reported in corals for the first time (Lee et al., 2012; Li et al., 2013), and the number of associated bacteria from one coral sample could reach more than 2000 on the species or OUT level (Sunagawa et al., 2010; Li et al., 2014). However, a comparative analysis of bacterial diversities associated with corals is lacking. So the characteristics of coral-associated bacteria in different coral species are poorly understood.

Coral thermal bleaching events occur frequently and have become more and more serious with global warming and frequent El Niño phenomena (Glynn, 1993; Edwards et al., 2001; Berkelmans et al., 2004; McDermott, 2016). However, different coral species have different tolerances to thermal stress. In 1998, large-scale coral colonies had been investigated on reefs fringing inshore islands on the Great Barrier Reef during a large-scale coral bleaching event. A detailed analysis showed that most of the branching corals (BC), such as *Acroporidae*, *Pocilloporidae*, and *Milleporidae* (*Isopora*, *Pocillopora damicornis*, *Stylophora pistillata*, and so on), were highly susceptible, while massive corals (MC) such as *Faviidae*, *Fungiidae*, *Oculinidae*, and so on (e.g., *Cyphastrea*, *Turbinaria*, and *Galaxea*) were relatively unaffected by bleaching stress (Marshall and Baird, 2000). The data on coral bleaching, subsequent mortality and community succession were collected from 1990 to 1998 on artificial and natural reefs in the Maldives (Edwards et al., 2001). Owing to sea surface temperature (SST) anomalies, approximately 98% of branching Pocilloporidae and Acroporidae on artificial structures deployed on a reef flat in 1990 died, whereas the most of massive Poritidae, Agariciidae, and Faviidae survived the bleaching event. The coral communities contained 95% BC and 5% MC before a bleaching event of the artificial reefs in 1994, and the post-bleaching communities consisted of 3% BC and 97% MC. These results were also confirmed in a laboratory simulation experiment. For example, the response of nine species of stony coral (*Acropora* spp., *Pocillopora damicornis*, *Pavona decussate*, *P. lutea*, and *Montipora* sp.) from Luhuitou fringing reef to high temperature treatments (ranging from 26 to 32°C) was surveyed (Li et al., 2008). The results showed that BC of *Acropora* spp. and *P. damicornis* were most susceptible to bleaching, while the MC *P. decussate*, *P. lutea*, and *Montipora* sp. had better endurance to high temperature (32°C).

In contrast to coral thermal bleaching, cold bleaching will occur when the SST is abnormally low (Saxby et al., 2003; Hoegh-Guldberg and Fine, 2004; Yu et al., 2004; Yu, 2012). In this case, MC also show stronger tolerance to cold stress than BC. In 2003, large-scale branching *Acroporidae* exposed at low tide bleached and died because of extremely cold temperatures (12°C) at Heron Island, on the southern end of the Great Barrier Reef (Hoegh-Guldberg and Fine, 2004). In laboratory experiments, the tolerance and response of different coral species in low water temperature (ranged from 24 to 14°C) showed similar results: (Li et al., 2009) the branching *Acropora* spp. died most easily, while massive *P. lutea* and *P. decussate* had a significantly higher tolerance of low water temperature.

It is well known that coral-associated bacteria play an important role in the coral holobiont, and show extremely high diversity. Furthermore, different coral species have different tolerances to thermal or cold stress. However, the relationship between coral-associated bacteria and coral susceptibility to thermal or cold stress are currently unclear. Some researchers think that coral bleaching tolerance is related to *Symbiodinium* density because previous studies had revealed that MC generally had a higher algal density than the BC. In addition, the coral-associated bacterial communities were structured by multiple factors at different scales. Coral bleaching events caused by high or low temperature have occurred frequently in the South China Sea (Yu, 2012), which is a natural laboratory for studying the response of coral reefs to extreme climate events as well as evolutionary characteristics of coral. In this study, a certain amount of scale massive and BC were used to analyze bacterial diversity; the corals were collected at the same time and location (Xinyi Reef of the South China Sea), to control for the influences of geography, season, and some environmental factors. The main purpose is to explore the characteristics of bacterial diversities associated with different coral species, and further explore the relationship between coral-associated bacteria and coral susceptibility to thermal or cold stress.

## Materials and Methods

### Study Site, Sample Collection, and Species Identification

**FIGURE 1:**
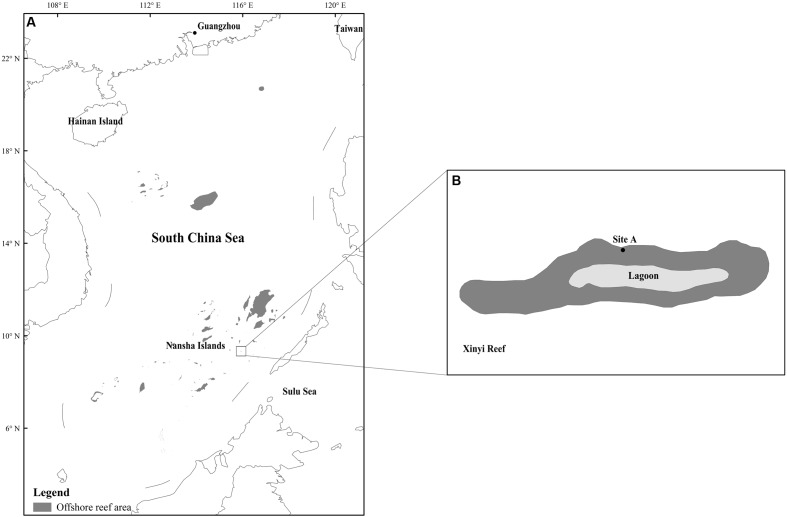
Location map of sampling site A at Xinyi Reef, South China Sea. **(A)** Map of the whole South China Sea with labeled coral reef area. The Xinyi Reef is marked by a box. **(B)** The Xinyi Reef is magnified showing sampling site A (9°20′6″ N, 115°55′49″ E). The map was constructed using software ArcGIS (ver. 10.1). The offshore reef area was drawn using remote sensing images (fused by landsat 8 multispectral bands and panchromatic band) with resolution of 15 m.

Our study site is located at Xinyi Reef of Nansha Islands, in the South China Sea. In May 2015, coral samples were collected using a hammer and chisel within 100 m and at a depth of 9–10 m of site A (9°20′6″ N, 115°55′49″ E) at Xinyi Reef (**Figure 1**). The SST, pH value and salinity were approximately 31.2°C, 8.21 and 33.1, respectively. Three replicated samples (approximately 8 cm × 8 cm) per coral species were collected from the side of the colonies. The distance between two repeated samples is above 10 m. As many different coral species as possible were collected. The collected samples were washed with sterile seawater and then placed in sterile plastic bags. All samples were briefly stored at low temperatures (0–4°C) and then immediately transported back to the laboratory for DNA extraction.

Coral tissue was removed using airbrush and coral skeleton was immediately ready for each species identification. All coral samples were identified according to their ecological and morphological characteristics.

### DNA Extraction, PCR Amplification, and Illumina MiSeq Sequencing

Small pieces of coral sample including tissue, mucus and skeleton (approximately 50 mg), cut with a pair of scissors, were used to extract genomic DNA using TIANamp Marine Animals DNA Kit [Tiangen Biotech (Beijing) Co., Ltd., Beijing, China] according to the manufacturer’s instructions. The V3–V4 region of bacterial 16S rRNA gene was amplified using the bacterial-specific forward primer 338F (5′-ACTCCTACGGGAGGCAGCAG-3′) and reverse primer 806R (5′-GGACTACHVGGGTWTCTAAT-3′), where barcode is an eight-base sequence unique to each sample (Mori et al., 2014; Xu et al., 2016). The reaction system and procedure of PCR using a ABI GeneAmp^®^ 9700 thermal cycler are the same as previously described (Sun et al., 2014). Triplicate PCR products were pooled for each sample, and then fragments with size in the range of 421–460 bp were purified and quantified using the AxyPrep DNA gel extraction kit (Axygen Biosciences, Union City, CA, United States) and QuantiFluor^TM^-ST Fluorescence quantitative system (Promega, United States). Purified amplicons were pooled in equimolar amounts and paired-end sequenced (2 × 250) on an Illumina MiSeq platform according to the standard protocols (Majorbio Bio-Pharm Technology Co. Ltd., Shanghai, China). The raw reads were deposited into the NCBI Sequence Read Archive (SRA) database (Submission Number: SUB2295385).

### Data Analysis

Raw sequences were optimized using the software platform Trimmomatic to exclude the reads with homopolymer inserts and low quality scores (<20) (Bolger et al., 2014). The remaining high-quality reads were used for taxonomic analysis. After removing the chimeric sequences, all reads were identified and classified using the Ribosomal Database Project (RDP) by setting a bootstrap confidence level of 70% to cluster into operational taxonomic units (OTUs) at a 97% identity threshold and obtain the representative sequence for each OTU (Edgar, 2010). Based on the OTUs cluster analysis, a series of statistical analysis indices, including Coverage, ACE estimator, Shannon index, and so on, were calculated for each sample using the software Mothur (version v.1.30.1) (Schloss et al., 2011). Taxonomy was aligned and compared with the SILVA database^1^ (Release 123) (Quast et al., 2013) using the Qiime platform^2^. The beta diversity analysis was executed using the principal co-ordinates analysis (PCoA) at the OTU level.

## Results

### Identification of Coral Species and Skeletal Morphology Analysis

A total of 25 scleractinian coral samples were selected to test for symbiotic bacteria. These coral samples were identified as belonging to 6 families, 10 genera, and 18 species based on their ecological and morphological characteristics (**Table 1**). They were then divided into two groups according to skeletal morphology: 16 MC and 9 BC. Massive corals included Faviidae, Poritidae, Fungiidae, and Mrulinidae, while BC included Acroporidae and Pocilloporidae.

**Table 1:** Coral species identification.

Index		Family	Genus	Species
Massive coral	M01	Faviidae	*Goniastrea*	*Goniastrea edwardsi*
	M02	Faviidae	*Plesiastrea*	*Plesiastrea curta*
	M03	Faviidae	*Leptastrea*	*Leptastrea bottae*
	M04	Faviidae	*Leptastrea*	*Leptastrea bottae*
	M05	Faviidae	*Leptastrea*	*Leptastrea* sp.
	M06	Faviidae	*Platygyra*	*Platygyra daedalea*
	M07	Faviidae	*Platygyra*	*Platygyra crosslandi*
	M08	Faviidae	*Platygyra*	*Platygyra crosslandi*
	M09	Faviidae	*Echinopora*	*Echinopora gemmacea*
	M10	Poritidae	*Porites*	*Porites rus*
	M11	Poritidae	*Porites*	*Porites rus*
	M12	Poritidae	*Porites*	*Porites lutea*
	M13	Poritidae	*Porites*	*Porites lutea*
	M14	Fungiidae	*Fungia*	*Fungia scutaria*
	M15	Fungiidae	*Fungia*	*Fungia fungites*
	M16	Mrulinidae	*Leptoria*	*Leptoria phrygia*
Branching coral	B01	Acroporidae	*Acropora*	*Acropora brueggemanni*
	B02	Acroporidae	*Acropora*	*Acropora brueggemanni*
	B03	Acroporidae	*Acropora*	*Acropora humilis*
	B04	Acroporidae	*Acropora*	*Acropora* sp.
	B05	Pocilloporidae	*Pocillopora*	*Pocillopora eydouxi*
	B06	Pocilloporidae	*Pocillopora*	*Pocillopora eydouxi*
	B07	Pocilloporidae	*Pocillopora*	*Pocillopora eydouxi*
	B08	Pocilloporidae	*Pocillopora*	*Pocillopora* sp.
	B09	Pocilloporidae	*Pocillopora*	*Pocillopora verrucosa*


### Diversity of Coral-Associated Bacteria

The number of recovered reads was no less than 30,460 for each sample after quality filtering; these were clustered into different 97% OTUs. The length of those sequences ranged from 421 to 460 bp. The Good’s coverage of each sample library was more than 99%. These sequencing results thus represented the true condition of the bacteria in the sample. Other statistical analysis indices including ACE, Chao, and Shannon are listed in **Table 2**. These indices showed an obvious difference between MC and BC, with community diversity higher in MC than that in the BC. The number of OTUs was also generally higher in MC than branching. Among them, there are six samples with greater than 1000 OTUs including M02, M05, etc. Contrarily, the number of OTUs was lower than 700 from all the BC (**Table 2**). The Shannon indices are similar in all MC but higher than in BC. This means that the diversity and equitability of bacteria associated with MC are higher than BC. However, they also show that a few MC are relatively low (1.91 in M04, 3.53 in M06, and 3.17 in M16) compared to the other massive samples (5.81 in M02, 5.88 in M09, etc.). The value of the Shannon index in BC ranged from 2.28 to 4.89.

**Table 2:** Numbers of sequences and operational taxonomic units (OTUs) (97%) and diversity estimates of bacteria associated with different morphologic corals.

Index		No. of Seq	OTUs	ACE	Chao	Coverage	Shannon
Massive coral	M01	41680	766	794.87	836.04	0.998412	5.21
	M02	35444	1416	1500.66	1543.55	0.994396	5.81
	M03	31922	624	659.02	681.78	0.997869	4.68
	M04	39438	410	422.98	430.32	0.999059	1.91
	M05	39070	1277	1345.71	1361.36	0.995907	5.63
	M06	33930	729	758.92	760.36	0.997774	3.53
	M07	36843	965	1044.91	1056.97	0.996263	5.28
	M08	43063	639	659.84	669.75	0.998929	5.17
	M09	42064	1262	1320.19	1360.11	0.996746	5.88
	M10	33750	1274	1352.08	1389.64	0.994473	5.64
	M11	40612	568	581.87	587.50	0.999298	5.21
	M12	41705	1186	1410.66	1443.40	0.99196	5.71
	M13	31995	1090	1258.90	1274.29	0.992344	5.59
	M14	37203	544	557.94	569.38	0.999094	4.97
	M15	39468	955	1021.05	1038.38	0.996649	5.45
	M16	31134	492	539.49	547.86	0.997282	3.17
Branching coral	B01	30460	644	682.26	693.52	0.997745	4.81
	B02	34571	643	668.94	694.04	0.998468	4.89
	B03	35185	451	466.51	476.62	0.998879	3.85
	B04	40201	497	515.65	517.50	0.998926	3.50
	B05	34747	430	466.22	475.64	0.997978	2.28
	B06	31229	689	709.98	717.68	0.998239	4.58
	B07	39195	321	332.22	334.59	0.999414	3.82
	B08	44505	498	513.35	527.06	0.999273	4.52
	B09	35440	680	884.31	870.41	0.993343	2.77


### Bacterial Community Composition

**FIGURE 2:**
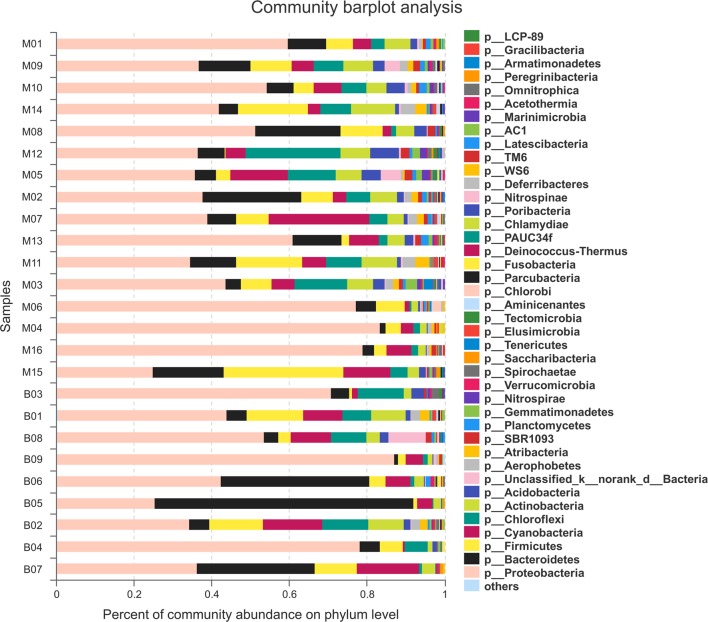
Bacterial composition profiles. Taxonomic classification of bacterial reads retrieved from different coral species at phylum level using RDP classifier. “others” represent the bacterial phyla with abundances of less than 0.01%.

Fifty-five bacterial phyla, including 43 formally described bacterial phyla and 12 candidate phyla, were recovered from the 25 coral samples (**Figure 2**). Among them, 43 bacterial phyla were present in both MC and BC groups. Nine bacterial phyla (including five candidate phyla) were unique groups to the MC sample libraries and 3 (including 2 candidate phyla) to BC, respectively (Supplementary Figure S1). According to the average value of abundance for each bacterial group, the dominant bacteria were similar to those previously reported, of which *Proteobacteria* (52.56%), *Bacteroidetes* (12.83%), *Firmicutes* (8.66%), *Cyanobacteria* (6.75%), and *Chloroflexi* (5.63%) were predominant in the all of sample libraries. The highest abundance of *Proteobacteria* reached 87.39, 83.78, and 79.81% in B09, M04. and M16 libraries, respectively. However, *Bacteroidetes* was the most predominant bacterial phylum in B05 (66.64%), B06 (37.98%), and B07 (30.73%) sample libraries relative to the all MC (ranged from 1.71 to 25.71%) and the other BC (1.11–5.24%). *Firmicutes* was the most predominant in M15 library (31.34%), though low abundance was detected in the other sample libraries (ranged from 0.67 to 17.89%). Additionally, *Cyanobacteria* and *Chloroflexi* were the second dominant bacterial phylum in M07 (25.99%) and M12 (24.40%) libraries, respectively.

At the class level, *Gammaproteobacteria* and *Alphaproteobacteria* were the most abundant group in most of the coral species libraries (Supplementary Figure S2). For example, the abundance of *Gammaproteobacteria* in M04, M06, and M16 could reach 74.42, 46.39, and 46.83%, respectively. *Alphaproteobacteria* were the most predominant bacterial class, and in M01, M13, and B09 could reach 38.98, 39.07, and 41.44%, respectively. On the other hand, it was unique that *Cytophagia* was the first dominant group in B05 (64.53%) and B06 (28.25%). Although the bacterial compositions exhibited high complexity at other taxonomic levels (order, family, genus, and species) (Supplementary Figures S3–S6), bacterial diversities associated with MC were generally higher than those in BC (**Table 3**). However, the complexity of few samples was lower, e.g., M04, B07, B05, and B03 (**Table 3** and Supplementary Figures S3–S6).

**Table 3:** Numbers of bacteria associated with different skeletal morphologic corals in different taxonomic levels.

Index		Phylum	Class	Order	Family	Genus	Species	Unclassified species
Massive coral	M01	26	61	138	222	335	486	280
	M02	40	84	182	292	497	751	665
	M03	31	60	139	204	317	450	174
	M04	29	54	109	154	247	318	92
	M05	32	77	173	277	433	676	601
	M06	26	57	126	207	338	467	262
	M07	37	77	163	263	427	601	364
	M08	20	47	107	173	296	408	231
	M09	38	80	169	286	494	725	537
	M10	38	83	183	304	488	721	553
	M11	29	57	129	202	332	412	156
	M12	26	62	127	192	290	501	685
	M13	29	66	133	221	354	538	552
	M14	28	55	125	201	329	414	130
	M15	28	59	136	235	437	607	348
	M16	27	55	118	192	311	387	105
Branching coral	B01	31	60	135	217	370	479	165
	B02	29	54	135	216	351	463	180
	B03	16	36	90	145	225	304	147
	B04	21	41	92	165	287	365	132
	B05	17	36	90	154	261	324	106
	B06	28	56	122	201	351	476	213
	B07	17	39	84	139	211	252	69
	B08	24	46	104	158	236	317	181
	B09	29	60	136	218	362	498	182


There were 532 families detected from the 25 corals samples, including 123 families in MC groups and 21 families in BC groups, as well as 388 families that were shared in both MC and BC groups. *Rhodobacteraceae*, *Hahellaceae*, *Flammeovirgaceae*, and *Vibrionaceae* were dominant bacteria groups with a higher average abundance of 11.40, 7.23, 6.51, and 3.38% in all coral samples, respectively (Supplementary Figure S7). Among them, *Rhodobacteraceae*, *Vibrionaceae*, and *Alteromonadaceae* were potential coral pathogens and had an average abundance of 11.40, 3.38, and <1.00%, respectively. However, confirmed coral pathogens were not detected from all coral species.

### Bacterial Composition Associated with Different Coral Species

**FIGURE 3:**
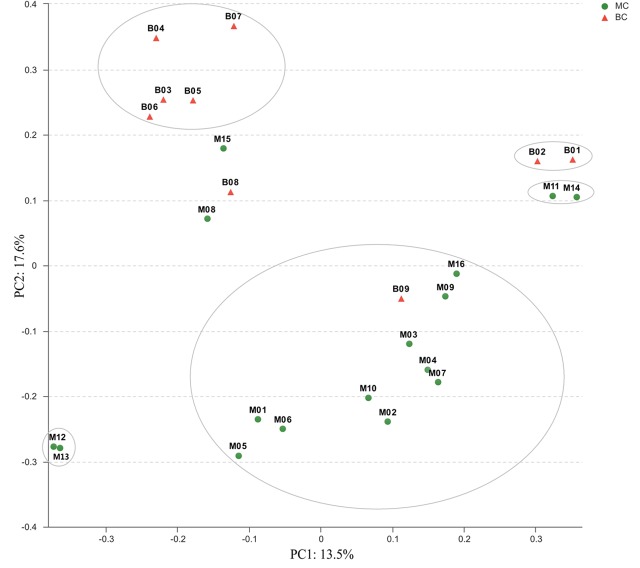
Principal co-ordinates analysis (PCoA) plot based on the OTU level from 25 coral species. The scatter plot is of principal coordinate 1 (PC1) vs. principal coordinate 2 (PC2). PC1 and PC2 represent the principal factors affecting bacterial composition associated with corals.

The similarity among the bacterial communities associated with 25 coral samples was evaluated using PCoA and hierarchical clustering analysis on OTU level. The PCoA analysis using the Bray–Curtis metric showed that coral samples with different bacterial communities could roughly cluster into two groups (**Figure 3**). Group I contained all of the MC species except the M08, M11, M12, M13, M14, and M15 samples. However, M11/M14 and M12/M13 samples were very closely clustered together. Group II mainly contained BC species. Similar results were also confirmed by the Hierarchical clustering trees analysis (Supplementary Figure S8). Clustering and PCoA results indicated that the most significant factor affecting bacterial species composition was skeletal morphology of the coral host.

## Discussion

### Coral Species Size and Bacterial Diversity Analysis

Presently, an ever-increasing number of scleractinian corals are being selected for surveys of bacterial diversity associated with coral (Sunagawa et al., 2010; Chen et al., 2011; Ceh et al., 2012; Lee et al., 2012; McKew et al., 2012; Li et al., 2013, 2014). These previously described coral species cover different genera (including *Isopora*, *Pocillopora*, *Astreopora*, *Stylophora*, *Acropora*, *Porites*, *Montastraea*, *Diploria*, *Gorgonia*, *Tubastrea*, etc.) and were collected from different locations (Caribbean Sea, Red Sea, South China Sea, Great Barrier Reef, Maldives, and so on). These studies indicate that the diversities of coral-associated bacteria were extremely high, and many bacterial phyla (*BRC1*, *OD1*, and *SR1*) were reported in corals for the first time (Li et al., 2013). However, the number of coral species for each study was small (less than 10 species) and the main attention of the bacterial-coral association surveys was focused on species specificity (Hong et al., 2009; Morrow et al., 2012), compartment specificity (Sweet et al., 2011; Li et al., 2014), geographic specificity (McKew et al., 2012), and season specificity (Hong et al., 2009; Chen et al., 2011; Li et al., 2014). In this study, large-scale coral samples involving 18 species, 10 genera, and 6 families were collected to analyze the bacterial communities in the Xinyi Reef of South China Sea (**Table 1**). These coral species could be clearly divided into two groups according to their skeletal morphology, which were massive and branching (Supplementary Material). Compared with previous studies, more bacterial phyla were detected from single coral libraries in this study. For example, the higher numbers of bacterial phyla associated with massive *Plesiastrea curta*, *Echinopora gemmacea*, *Porites rus* (M10), *Platygyra crosslandi* (M07), and *Leptastrea* sp. were 40, 38, 38, 37, and 32, respectively. And the bacterial phyla of other coral species ranged from 16 to 31 (most coral species > 25) (**Table 3**). While using the same sequencing method, Li et al. (2013) indicated that the bacterial diversity was higher in corals *Porites lutea* and *Galaxea fascicularis* from Sanya Bay in South China Sea than those from the other locations. In that study, 23 bacterial phyla were detected, which was higher than in previous studies (bacterial phyla < 20). Altogether, 55 bacterial phyla including 43 formally described bacterial phyla and 12 candidate phyla were recovered from the 25 coral samples in this study. Among them, plenty of bacterial phyla may represent phyla that have not been found in corals previously (**Figure 2**). Therefore, our study can provide more significant information in coral-associated bacteria based on the advantages of coral species size and bacterial diversities.

The most dominant bacteria were *Proteobacteria* in most of the coral species, followed by members of the *Bacteroidetes*, *Firmicutes*, *Cyanobacteria*, and *Chloroflexi*, which was similar to previous studies (Ceh et al., 2012; Lee et al., 2012; Li et al., 2013). The highest abundance of *Proteobacteria* could reach 87.39, 83.78, and 79.81% in B09, M04, and M16 libraries, respectively. However, *Bacteroidetes* was the most predominant bacteria in B05 (66.64%), B06 (37.98%), and B07 (30.73%) libraries relative to all the MC (ranged from 1.71 to 25.71%) and the other BC (1.11–5.24%); this comparison was reported for the first time in this study. In addition, *Firmicutes* was the most predominant in M15 library (31.34%), which was similar to that in the mucus and skeleton of *Porites lutea* collected in August in the Luhuitou fringing reef of the South China Sea (Li et al., 2014), but *Firmicutes* was rarely found in other previous studies. Furthermore, 12 candidate phyla, including *WS2*, *RBG-1L*, *CP-89*, and so on, were detected in all coral species. Many novel bacterial species are not annotated.

### The Relationship between Bacterial Diversity and Coral Potentially Tolerances to Thermal or Cold Stress

The bacterial diversities associated with corals from MC versus BC groups had significant characteristic differences. The bacterial diversities associated with MC groups were generally higher than those of BC groups at different taxonomic levels (**Table 3**). For example, the average values of number for bacterial phyla in MC and BC groups were 30 and 24, respectively. In addition, the average OTU level values were 887 and 539, respectively, in MC and BC groups. Although Li et al. (2013) indicated that MC (*Porites lutea* and *Porites lutea*) had higher estimated diversities than BC (*Acropora millepora*), only three coral species were analyzed. Therefore, this study further certified that coral skeletal morphology plays a key role in determining the diversities of bacteria associated with corals by increasing the number of coral species. Meanwhile, the more similar studies from other geographic locations are needed to prove this possible relationship.

This study also showed that the potentially tolerance of scleractinian coral to thermal or cold stress is related to its skeletal morphology. As described in the introduction, MC species have better tolerances to thermal or cold stress than BC species. Combining with the analysis of bacterial diversity in this study, it can be inferred that abundant bacterial species may help corals respond to abnormal changes of temperature. The mechanism is not clear, which may be related to the microbial regulation of coral reef ecosystems. Shinzato et al. (2011) completed the genome sequencing of *Acropora digitifera* and identified more than 20,000 protein-coding genes. This study not only disclosed the development age of coral, but also found that *Acropora* might have lost the essential gene of cysteine biosynthesis, thus be metabolically dependent on its symbionts. Such relationship made the coral particularly sensitive to the climate change which endangered its symbiotic. Lin et al. (2015) successfully decoded the genome of *Symbiodinium kawagutii* and identified protein-coding genes and *Symbiodinium*-specific gene families. From the genetic evidence, this study strongly demonstrated that *S. kawagutii* could alter their genetic structure in the history of symbiosis to suit for the environmental changes better. Metagenomic analysis of the microbial community associated with coral also provided some insights into the coral responding to environmental changes (Wegley et al., 2007). Therefore, more related studies in the future will be helpful in unraveling the mechanism of coral susceptibility to thermal or cold stress.

### Cluster Analysis Based on the Similarity of Coral-Associated Bacteria

Although the bacterial communities associated with different coral species were very complex, PCoA and hierarchical clustering analysis obviously showed that massive and branching corals could roughly cluster into different groups (**Figure 3** and Supplementary Figure S8). Comparing with previous studies, this study improved the number of coral species and classified samples according to their skeletal types, and then implemented clustering analysis based on the similarity of coral-associated bacteria. These results revealed that both MC and BC have their own associated bacteria, which will provide a theoretical basis for future studies of genetic evolution of coral holobionts.

### Potential Pathogens in Corals

Over the past 30 years, various coral diseases have caused severe damage to coral reefs worldwide. However, these coral diseases occurred mostly because environmental changes led to colonization and abnormal reproduction of pathogens in coral hosts (Rosenberg and Ben-Haim, 2002; Ben-Haim et al., 2003; Reshef et al., 2006; Rosenberg et al., 2007; Tout et al., 2015). Currently, the confirmed coral pathogens mainly include *Vibrio shiloi* (Kushmaro et al., 1996), *Vibrio coralliilyticus* (Ben-Haim et al., 2003), *Vibrio carchariae* (Ritchie and Smith, 1995), *Vibrio alginolyticus* (Cervino et al., 2004), *Aspergillus sydowii* (Geiser et al., 1998), *Aurantimonas coralicida* (Denner et al., 2003), *Thalassomonas loyana* (Barash et al., 2005), and *Serratia marcescens* (Patterson et al., 2002). Fortunately, none of these coral pathogens were detected from 924,854 reads in 25 coral sample libraries in this study. This suggests that these corals are less likely to be threatened by common bacterial diseases in this sea area. Sharon and Rosenberg (2010) indicated that *Vibrio* species in coral mucus are largely in the viable-but-non-culturable (VBNC) state, and may contribute to the health of corals by preventing infections from pathogens. In our study, the abundance of *Vibrionaceae* was also found to be high (average abundance of 3.38% in all coral species) (Supplementary Figure S7). This further suggests that *Vibrio* species are common in coral holobionts.

## Conclusion

Our results from this study showed that bacterial diversities associated with MC were generally higher than those associated with BC at different taxonomic levels. Furthermore, many bacterial phyla and unannotated bacterial species were detected in coral sample libraries. Combined with the differences in thermal or cold stress between massive and branching corals, we infer that abundant bacterial species can help corals respond to abnormal changes of temperature. Although the mechanism is not clear, this study provides novel insights into the relationship between the high complexity of bacterial community associated with coral, skeletal morphology of coral and potentially tolerances to thermal or cold stress.

## Author Contributions

KY and JL conceived the research, YW, XH, WH, and ZW contributed the materials, JL performed all experiments, ZQ and ZP drawn all pictures, QY and WW identified coral species, JL and KY wrote the manuscript, all authors edited and approved the manuscript.

## Conflict of Interest Statement

The authors declare that the research was conducted in the absence of any commercial or financial relationships that could be construed as a potential conflict of interest.

## Footnotes

1 http://www.arb-silva.de

2 http://qiime.org/scripts/assign_taxonomy.html

## References

[B1] Barash Y. Loya Y. Sulam R. Rosenberg E. (2005). Bacterial Strain BA-3 and a filterable factor cause a white plague-like disease in corals from the Eilat coral reef. *Aquat. Microb. Ecol.* 40 183–189. 10.3354/ame040183

[B2] Ben-Haim Y. Zicherman-Keren M. Rosenberg E. (2003). Temperature-regulated bleaching and lysis of the coral *Pocillopora damicornis* by the novel pathogen *Vibrio coralliilyticus*. *Appl. Environ. Microbiol.* 69 4236–4242. 10.1128/AEM.69.7.4236-4242.2003 12839805 PMC165124

[B3] Berkelmans R. De’ath G. Kininmonth S. Skirving W. J. (2004). A comparison of the 1998 and 2002 coral bleaching events on the Great Barrier Reef: spatial correlation, patterns, and predictions. *Coral Reefs* 23 74–83. 10.1007/s00338-003-0353-y

[B4] Bolger A. M. Lohse M. Usadel B. (2014). Trimmomatic: a flexible trimmer for Illumina sequence data. *Bioinformatics* 30 2114–2120. 10.1093/bioinformatics/btu170 24695404 PMC4103590

[B5] Bourne D. Iida Y. Uthicke S. Smith-Keune C. (2008). Changes in coral-associated microbial communities during a bleaching event. *ISME J.* 2 350–363. 10.1038/ismej.2007.112 18059490

[B6] Ceh J. Raina J. B. Soo R. M. Van K. M. Bourne D. G. (2012). Coral-bacterial communities before and after a coral mass spawning event on Ningaloo Reef. *PLoS ONE* 7:e36920 10.1371/journal.pone.0036920 PMC3353996 22629343

[B7] Cervino J. M. Hayes R. L. Polson S. W. Polson S. C. Goreau T. J. Martinez R. J. (2004). Relationship of *Vibrio* species infection and elevated temperatures to yellow blotch/band disease in Caribbean corals. *Appl. Environ. Microbiol.* 70 6855–6864. 10.1128/AEM.70.11.6855-6864.2004 15528553 PMC525219

[B8] Chen C.-P. Tseng C.-H. Chen C. A. Tang S.-L. (2011). The dynamics of microbial partnerships in the coral *Isopora palifera*. *ISME J.* 5 728–740. 10.1038/ismej.2010.151 20962876 PMC3105734

[B9] Denner E. B. Smith G. W. Busse H. J. Schumann P. Narzt T. Polson S. W. (2003). *Aurantimonas coralicida* gen. nov., sp. nov., the causative agent of white plague type II on Caribbean scleractinian corals. *Int. J. Syst. Evol. Microbiol.* 53 1115–1122. 10.1099/ijs.0.02359-0 12892136

[B10] Edgar R. C. (2010). Search and clustering orders of magnitude faster than BLAST. *Bioinformatics* 26 2460–2461. 10.1093/bioinformatics/btq461 20709691

[B11] Edwards A. J. Clark S. Zahir H. Rajasuriya A. Naseer A. (2001). Coral bleaching and mortality on artificial and natural reefs in Maldives in 1998, sea surface temperature anomalies and initial recovery. *Mar. Pollut. Bull.* 42 7–15. 10.1016/S0025-326X(00)00200-9 11382986

[B12] Fiore C. L. Jarett J. K. Olson N. D. Lesser M. P. (2010). Nitrogen fixation and nitrogen transformations in marine symbioses. *Trends Microbiol.* 18 455–463. 10.1016/j.tim.2010.07.001 20674366

[B13] Geiser D. M. Taylor J. W. RitchieandAmp K. B. Smith G. W. (1998). Cause of sea fan death in the West Indies. *Nature* 394 137–138. 10.1038/280799671296

[B14] Glasl B. Herndl G. J. Frade P. R. (2016). The microbiome of coral surface mucus has a key role in mediating holobiont health and survival upon disturbance. *ISME J.* 10 2280–2292. 10.1038/ismej.2016.9 26953605 PMC4989324

[B15] Glynn P. W. (1993). Coral reef bleaching: ecological perspectives. *Coral Reefs* 12 1–17. 10.1007/BF00303779

[B16] Hoegh-Guldberg O. Fine M. (2004). Low temperatures cause coral bleaching. *Coral Reefs* 23:444 10.1007/s00338-004-0401-2

[B17] Hong M.-J. Yu Y.-T. Chen C. A. Chiang P.-W. Tang S.-L. (2009). Influence of species specificity and other factors on bacteria associated with the coral *Stylophora pistillata* in Taiwan. *Appl. Environ. Microbiol.* 75 7797–7806. 10.1128/AEM.01418-09 19854921 PMC2794093

[B18] Kushmaro A. Loya Y. Fine M. Rosenberg E. (1996). Bacterial infection and coral bleaching. *Nature* 380:396 10.1038/380396a0

[B19] Lee O. O. Yang J. Bougouffa S. Wang Y. Batang Z. Tian R. (2012). Spatial and species variations in bacterial communities associated with corals from the Red Sea as revealed by pyrosequencing. *Appl. Environ. Microbiol.* 78 7173–7184. 10.1128/AEM.01111-12 22865078 PMC3457102

[B20] Lesser M. P. Mazel C. H. Gorbunov M. Y. Falkowski P. G. (2004). Discovery of symbiotic nitrogen-fixing cyanobacteria in corals. *Science* 305 997–1000. 10.1126/science.1099128 15310901

[B21] Li J. Chen Q. Long L.-J. Dong J.-D. Yang J. Zhang S. (2014). Bacterial dynamics within the mucus, tissue and skeleton of the coral *Porites lutea* during different seasons. *Sci. Rep.* 4:7320 10.1038/srep07320 PMC4256709 25475855

[B22] Li J. Chen Q. Zhang S. Huang H. Yang J. Tian X.-P. (2013). Highly heterogeneous bacterial communities associated with the South China Sea reef corals *Porites lutea*, *Galaxea fascicularis* and *Acropora millepora*. *PLoS ONE* 8:e71301 10.1371/journal.pone.0071301 PMC3737133 23940737

[B23] Li S. Yu K. Shi Q. Chen T. Zhao M. (2009). Low water temperature tolerance and responding mode of scleractinian corals in Sanya Bay. *Chin. J. Appl. Ecol.* 20 2289–2295. 20030156

[B24] Li S. Yu K. Shi Q. Chen T. Zhao M. Yan H. (2008). Experimental study of stony coral response to the high temperature in Luhuitou of Hainan Island. *Trop. Geogr.* 28 534–539.

[B25] Lin S. Cheng S. Song B. Zhong X. Lin X. Li W. (2015). The *Symbiodinium kawagutii* genome illuminates dinoflagellate gene expression and coral symbiosis. *Science* 350 691–694. 10.1126/science.aad0408 26542574

[B26] Mahmoud H. M. Kalendar A. A. (2016). Coral-associated actinobacteria: diversity, abundance, and biotechnological potentials. *Front. Microbiol.* 7:204 10.3389/fmicb.2016.00204 PMC4770044 26973601

[B27] Mao-Jones J. Ritchie K. B. Jones L. E. Ellner S. P. (2010). How microbial community composition regulates coral disease development. *PLoS Biol.* 8:e1000345 10.1371/journal.pbio.1000345 PMC2846858 20361023

[B28] Marshall P. A. Baird A. H. (2000). Bleaching of corals on the Great Barrier Reef: differential susceptibilities among taxa. *Coral Reefs* 19 155–163. 10.1007/s003380000086

[B29] McDermott A. (2016). Coral bleaching event is longest on record. *Science News*, June 22, 2016.

[B30] McKew B. A. Dumbrell A. J. Daud S. D. Hepburn L. Thorpe E. Mogensen L. (2012). Characterization of geographically distinct bacterial communities associated with coral mucus produced by *Acropora* spp. and *Porites* spp. *Appl. Environ. Microbiol.* 78 5229–5237. 10.1128/AEM.07764-11 22636010 PMC3416423

[B31] Meron D. Atias E. Kruh L. I. Elifantz H. Minz D. Fine M. (2011). The impact of reduced pH on the microbial community of the coral *Acropora eurystoma*. *ISME J.* 5 51–60. 10.1038/ismej.2010.102 20668489 PMC3105665

[B32] Mori H. Maruyama F. Kato H. Toyoda A. Dozono A. Ohtsubo Y. (2014). Design and experimental application of a novel non-degenerate universal primer set that amplifies prokaryotic 16S rRNA genes with a low possibility to amplify eukaryotic rRNA genes. *DNA Res.* 21 217–227. 10.1093/dnares/dst052 24277737 PMC3989492

[B33] Morrow K. M. Moss A. G. Chadwick N. E. Liles M. R. (2012). Bacterial associates of two Caribbean coral species reveal species-specific distribution and geographic variability. *Appl. Environ. Microbiol.* 78 6438–6449. 10.1128/AEM.01162-12 22773636 PMC3426691

[B34] Patterson K. L. Porter J. W. Ritchie K. B. Polson S. W. Mueller E. Peters E. C. (2002). The etiology of white pox, a lethal disease of the Caribbean elkhorn coral, *Acropora palmata*. *Proc. Natl. Acad. Sci. U.S.A.* 99 8725–8730. 10.1073/pnas.092260099 12077296 PMC124366

[B35] Quast C. Pruesse E. Yilmaz P. Gerken J. Schweer T. Yarza P. (2013). The SILVA ribosomal RNA gene database project: improved data processing and web-based tools. *Nucl. Acids Res.* 41 D590–D596. 10.1093/nar/gks1219 23193283 PMC3531112

[B36] Reshef L. Koren O. Loya Y. Zilber-Rosenberg I. Rosenberg E. (2006). The coral probiotic hypothesis. *Environ. Microbiol.* 8 2068–2073. 10.1111/j.1462-2920.2006.01148.x 17107548

[B37] Ritchie K. B. Smith G. W. (1995). Preferential carbon utilization by surface bacterial communities from water mass, normal, and white-band diseased *Acropora cervicornis*. *Mol. Mar. Biol. Biotechnol.* 4 345–352.

[B38] Rosenberg E. Ben-Haim Y. (2002). Microbial diseases of corals and global warming. *Environ. Microbiol.* 4 318–326. 10.1046/j.1462-2920.2002.00302.x 12071977

[B39] Rosenberg E. Koren O. Reshef L. Efrony R. Zilber-Rosenberg I. (2007). The role of microorganisms in coral health, disease and evolution. *Nat. Rev. Microbiol.* 5 355–362. 10.1038/nrmicro1635 17384666

[B40] Saxby T. A. Dennison W. C. Hoegh-Guldberg O. (2003). Photosynthetic responses of the coral *Montipora digitata* to cold temperature stress. *Mar. Ecol. Prog. Ser.* 248 85–97. 10.3354/meps248085

[B41] Schloss P. D. Gevers D. Westcott S. L. (2011). Reducing the effects of PCR amplification and sequencing artifacts on 16S rRNA-based studies. *PLoS ONE* 6:e27310 10.1371/journal.pone.0027310 PMC3237409 22194782

[B42] Sharon G. Rosenberg E. (2010). Healthy corals maintain *Vibrio* in the VBNC state. *Environ. Microbiol. Rep.* 2 116–119. 10.1111/j.1758-2229.2009.00113.x 23766005

[B43] Shinzato C. Shoguchi E. Kawashima T. Hamada M. Hisata K. Tanaka M. (2011). Using the *Acropora digitifera* genome to understand coral responses to environmental change. *Nature* 476 320–323. 10.1038/nature10249 21785439

[B44] Sun Z. Li G. Wang C. Jing Y. Zhu Y. Zhang S. (2014). Community dynamics of prokaryotic and eukaryotic microbes in an estuary reservoir. *Sci. Rep.* 4:6966 10.1038/srep06966 PMC4225533 25382138

[B45] Sunagawa S. Woodley C. M. Medina M. (2010). Threatened corals provide underexplored microbial habitats. *PLoS ONE* 5:e9554 10.1371/journal.pone.0009554 PMC2832684 20221265

[B46] Sweet M. J. Croquer A. Bythell J. C. (2011). Bacterial assemblages differ between compartments within the coral holobiont. *Coral Reefs* 30 39–52. 10.1007/s00338-010-0695-1

[B47] Tout J. Siboni N. Messer L. F. Garren M. Stocker R. Webster N. S. (2015). Increased seawater temperature increases the abundance and alters the structure of natural *Vibrio* populations associated with the coral *Pocillopora damicornis*. *Front. Microbiol.* 6:432 10.3389/fmicb.2015.00432 PMC4435422 26042096

[B48] Wegley L. Edwards R. Rodriguez-Brito B. Liu H. Rohwer F. (2007). Metagenomic analysis of the microbial community associated with the coral *Porites astreoides*. *Environ. Microbiol.* 9 2707–2719. 10.1111/j.1462-2920.2007.01383.x 17922755

[B49] Xu N. Tan G. Wang H. Gai X. (2016). Effect of biochar additions to soil on nitrogen leaching, microbial biomass and bacterial community structure. *Eur. J. Soil Biol.* 74 1–8. 10.1016/j.ejsobi.2016.02.004

[B50] Yu K. (2012). Coral reefs in the South China Sea: their response to and records on past environmental changes. *Sci. Chin. Ear. Sci.* 55 1217–1229. 10.1007/s11430-012-4449-5

[B51] Yu K.-F. Zhao J.-X. Liu T.-S. Wei G.-J. Wang P.-X. Collerson K. D. (2004). High-frequency winter cooling and reef coral mortality during the Holocene climatic optimum. *Earth Planet. Sci. Lett.* 224 143–155. 10.1016/j.epsl.2004.04.036

