# Supplementary material for: Distinct Bacterial Communities Associated with Massive and Branching Scleractinian Corals and Potential Linkages to Coral Susceptibility to Thermal or Cold Stress
Source: Front Microbiol. 2017 Jun 8;8:979. doi: 10.3389/fmicb.2017.00979 (PMC5462945; doi:10.3389/fmicb.2017.00979)

**A close relationship between coral-associated bacteria and coral tolerance to thermal/cold stresses**

***Jiayuan Liang^1-3^, Kefu Yu^1-3*^, Yinghui Wang^1-3^, Xueyong Huang^1-3^, Wen Huang^1-3^, Zhenjun Qin^1-3^, Ziliang Pan^1-3^, Qiucui Yao^1-3^, Wenhuan Wang^1-3^, Zhengchao Wu^4^***

*^*^ Corresponding author, E-mail address: kefuyu@scsio.ac.cn (K. Yu)*

*^1^ Coral Reef Research Center of China, Guangxi University, Nanning 530004, China.*

*^2^ Guangxi Laboratory on the Study of Coral Reefs in the South China Sea, Nanning 530004, China*

*^3^ School of Marine Sciences, Guangxi University, Nanning 530004, China*

*^4^* *State Key Laboratory of Tropical Oceanography (LTO),* *South China Sea Institute of Oceanology,* *Chinese Academy of Sciences,* *Guangzhou 510301, China*

**Coral samples without tissue**

M01

M02

M03

M04

M05

M06

M07

M08

M09

M10

M11

M12

M13

M14

M15

M16

B01

B02

B03

B04

B05

B06

B07

B08


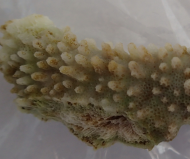


B09

**Figure S1** The distribution of bacterial phyla between massive and branching coral groups.

**
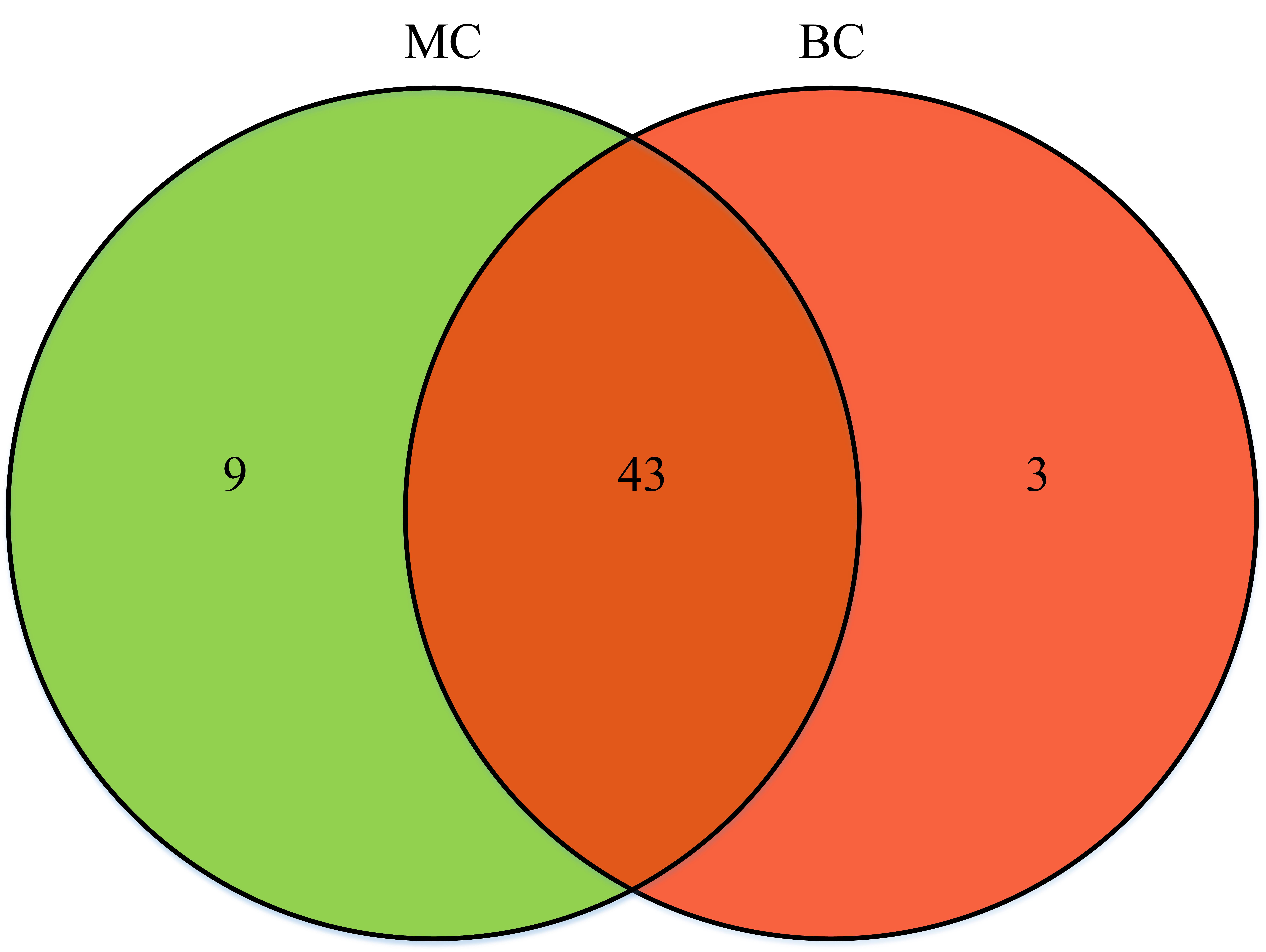
**

**Figure S2** Bacterial composition profiles. Taxonomic classification of bacterial reads retrieved from different coral species at class level using RDP classifier. “others” represent the bacterial class with abundance of less than 0.1%.


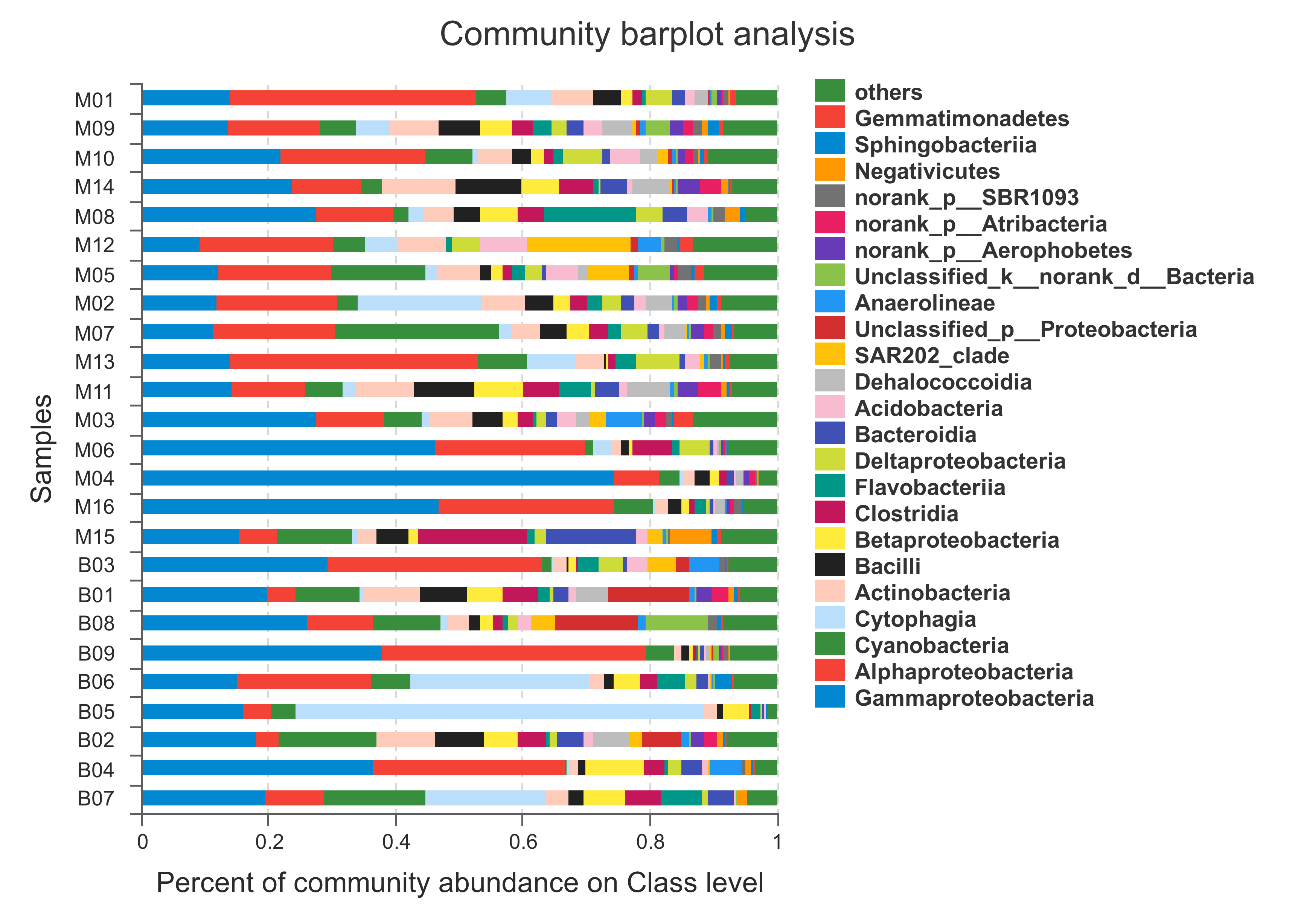


**Figure S3** Bacterial composition profiles. Taxonomic classification of bacterial reads retrieved from different coral species at order level using RDP classifier. “others” represent the bacterial order with abundance of less than 0.1%.


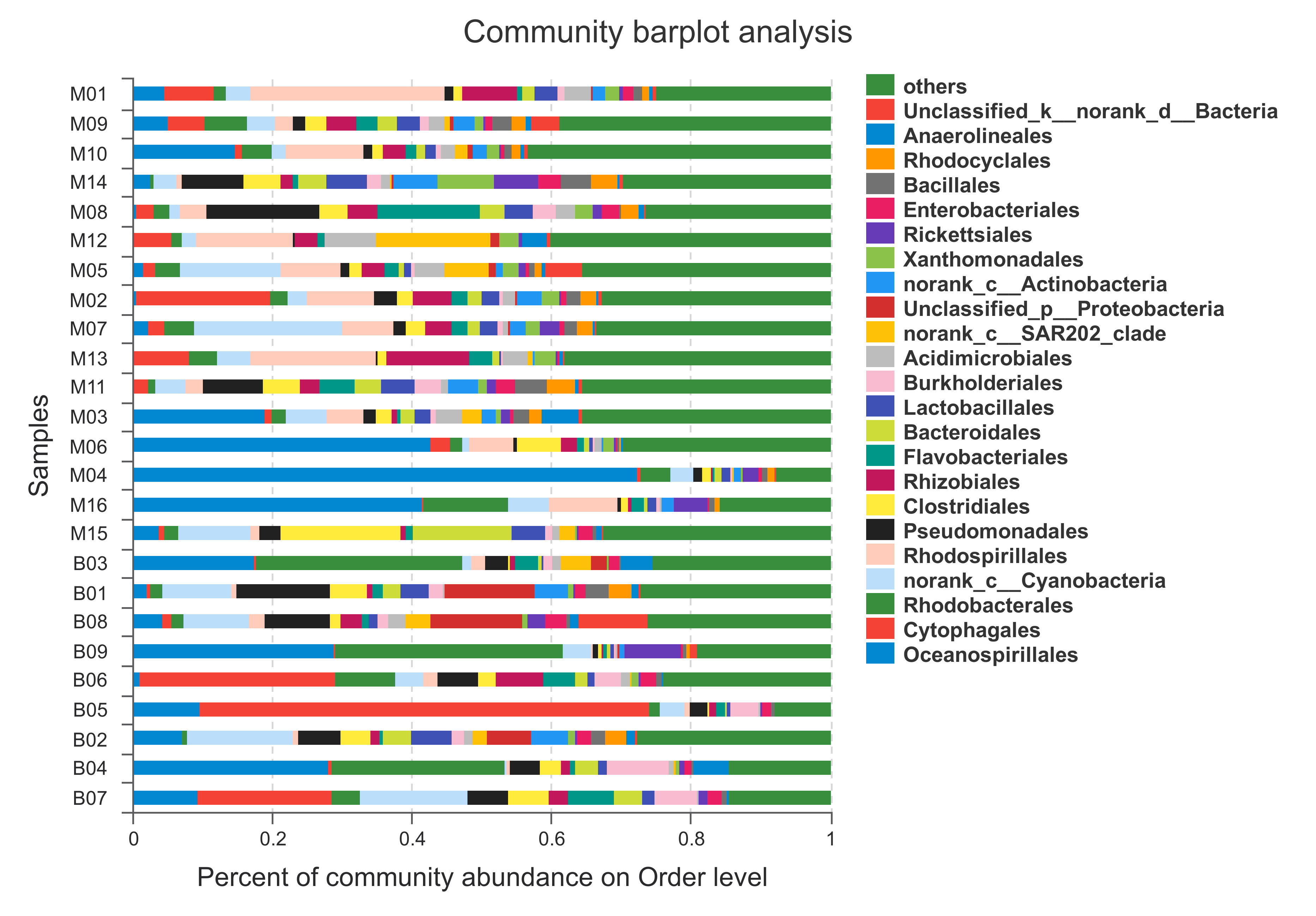


**Figure S4** Bacterial composition profiles. Taxonomic classification of bacterial reads retrieved from different coral species at family level using RDP classifier. “others” represent the bacterial family with abundance of less than 1%.


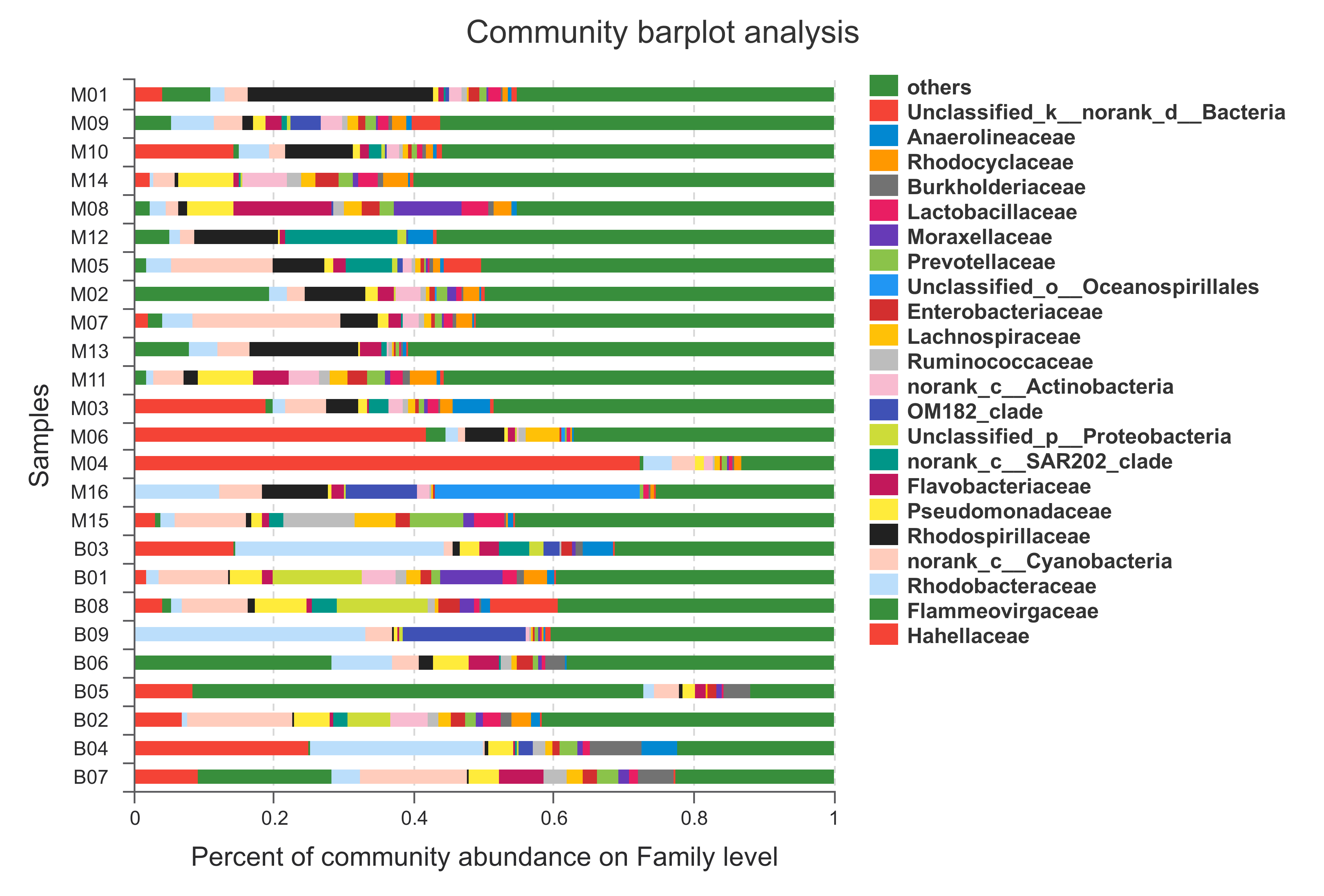


**Figure S5** Bacterial composition profiles. Taxonomic classification of bacterial reads retrieved from different coral species at genus level using RDP classifier. “others” represent the bacterial genus with abundance of less than 1%.


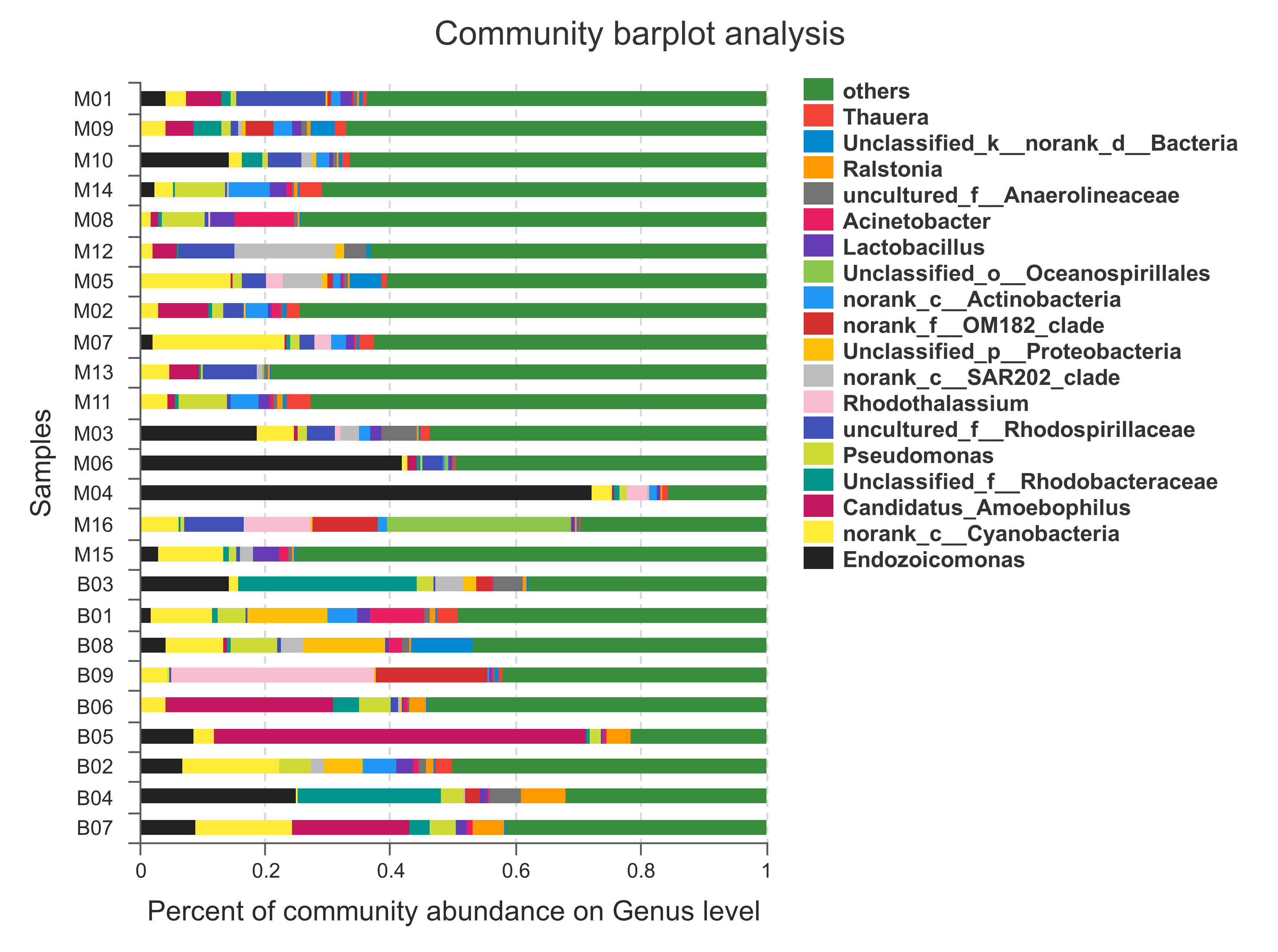


**Figure S6** Bacterial composition profiles. Taxonomic classification of bacterial reads retrieved from different coral species at species level using RDP classifier. “others” represent the bacterial species with abundance of less than 1%.


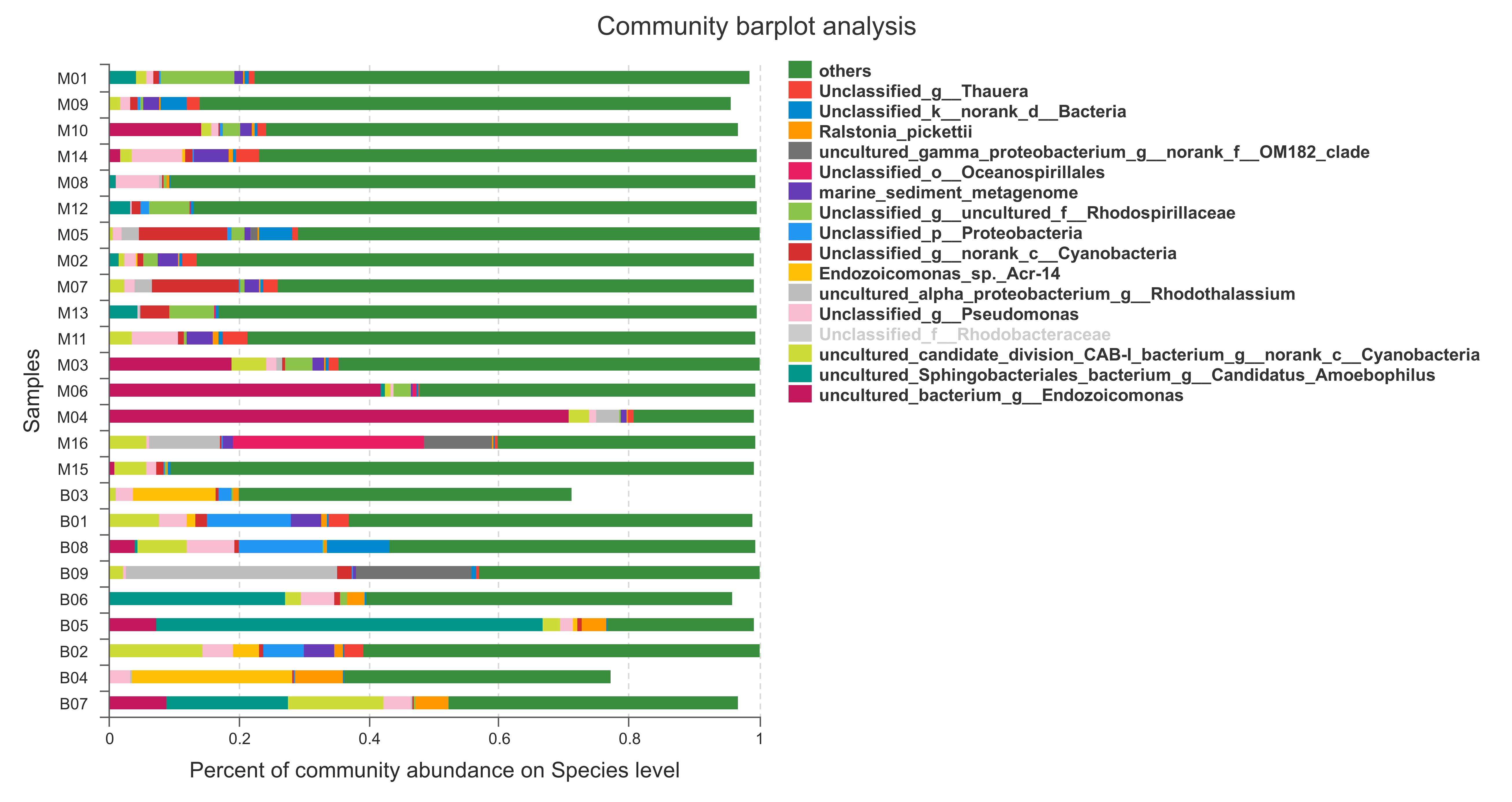


**Figure S7** Bacterial composition profiles. The average abundance of dominant bacteria in 25 coral species at family level. “others” represent the bacterial species with abundance of less than 1%.


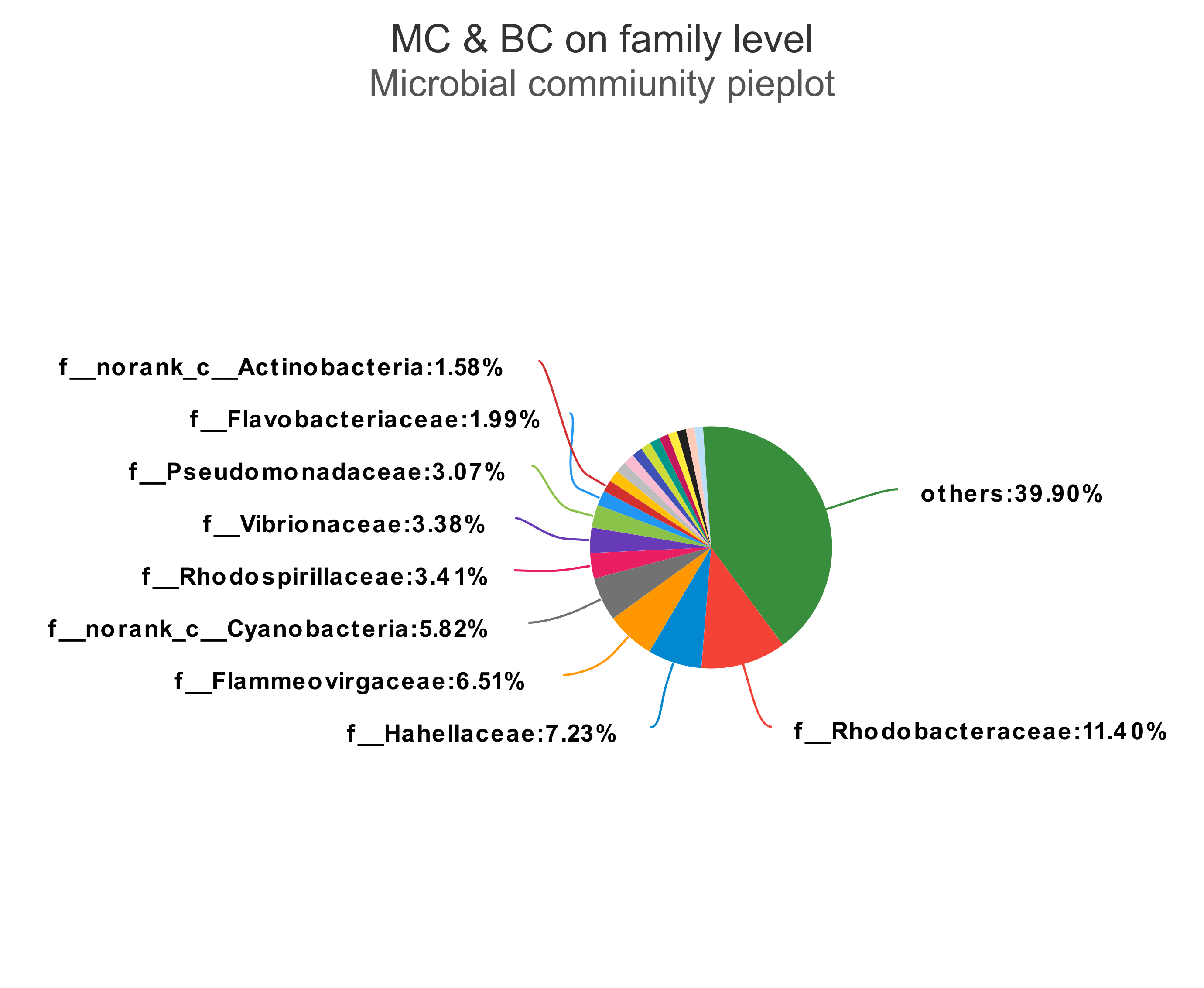

Supplement: Supplementary file 1 [file Data_Sheet_1.DOC]
